# Seeing eye to eye: trustworthy embodiment for task-based conversational agents

**DOI:** 10.3389/frobt.2023.1234767

**Published:** 2023-08-30

**Authors:** David A. Robb, José Lopes, Muneeb I. Ahmad, Peter E. McKenna, Xingkun Liu, Katrin Lohan, Helen Hastie

**Affiliations:** ^1^ Department of Computer Science, Heriot-Watt University, Edinburgh, United Kingdom; ^2^ Semasio, Porto, Portugal; ^3^ Department of Computer Science, Swansea University, Swansea, United Kingdom; ^4^ Department of Psychology, Heriot-Watt University, Edinburgh, United Kingdom; ^5^ Eastern Switzerland University of Applied Sciences, Buchs SG, Switzerland

**Keywords:** conversational agent, remote robots, autonomous systems, human–robot teaming, social robotics, user engagement, cognitive load

## Abstract

Smart speakers and conversational agents have been accepted into our homes for a number of tasks such as playing music, interfacing with the internet of things, and more recently, general chit-chat. However, they have been less readily accepted in our workplaces. This may be due to data privacy and security concerns that exist with commercially available smart speakers. However, one of the reasons for this may be that a smart speaker is simply too abstract and does not portray the social cues associated with a trustworthy work colleague. Here, we present an in-depth mixed method study, in which we investigate this question of embodiment in a serious task-based work scenario of a first responder team. We explore the concepts of trust, engagement, cognitive load, and human performance using a humanoid head style robot, a commercially available smart speaker, and a specially developed dialogue manager. Studying the effect of embodiment on trust, being a highly subjective and multi-faceted phenomena, is clearly challenging, and our results indicate that potentially, the robot, with its anthropomorphic facial features, expressions, and eye gaze, was trusted more than the smart speaker. In addition, we found that embodying a conversational agent helped increase task engagement and performance compared to the smart speaker. This study indicates that embodiment could potentially be useful for transitioning conversational agents into the workplace, and further *in situ*, “in the wild” experiments with domain workers could be conducted to confirm this.

## 1 Introduction

Conversational agents (CAs) are becoming ubiquitous in our homes^1^ with smart speakers, such as Amazon Alexa and Google Home, and virtual assistants on smart devices, such as Apple’s Siri. These home CAs tend to be used for simple transactional purposes such as setting a timer, accessing cooking recipes, and turning on lights. CAs are, however, increasingly used in more meaningful support roles, such as student debt advisor or managing harassment concerns in the workplace (Crockett et al., 2011).

The benefits of CAs include that users do not need to learn to use them (as they can communicate in natural language) and that users are often more willing to open up with a CA than with a human advisor as they are less afraid of being judged (Crockett et al., 2011). Despite research demonstrating such potential benefits of CAs, they are yet to be fully integrated into the workplace. We believe that their deployment could potentially result in enhanced worker engagement, performance, and efficiency, by streamlining maintenance operations. For example, CAs can control robots to carry out inspection tasks in safety critical settings (offshore renewable energy or nuclear environments), facilitating improved worker safety and also increasing productivity.

Giving a CA an embodied form, either virtual or physical, has been investigated as a way to enhance the interaction between the user and the agent and assist in forming an effective working relationship. For example, interacting with an embodied virtual assistant has been shown to result in lower task load compared to no embodiment (Kim et al., 2020). Furthermore, providing a virtual agent with a face in a facilitation task context has been shown to improve user trust, compared to voice-only interaction (Kim et al., 2018). In the context of a virtual estate agent, using a virtual embodiment improved conversational interaction such as turn-taking (Cassell et al., 2000). However, the benefits of CA embodiment may be quite nuanced and dependent on the task context, as described in the work of Kim et al. (2018) and Shamekhi et al. (2018).

In the work presented here, we are specifically interested in investigating the effects of CA embodiment on trust, user engagement, cognitive load (CL), and the quality of interaction for users performing a work-related task. CL, sometimes termed task load, refers to the burden placed on the user’s working memory, often called short-term memory, during a task (Paas et al., 2003). Going forward, we refer to the CA in this work as a mediator CA as it assists the user in a task of managing a small team of simulated robots. We simulated a working environment, creating a task that is both high-stakes, in terms of safety, and has a time-critical aspect. We created a simulated offshore energy installation populated by a team of mobile ground and aerial robots. Then, we developed simulated high-stakes emergency response scenarios, which would put participants in a challenging situation. In an experiment described here, we manipulated embodiment of the mediator CA in the following two conditions: embodied as a human-like robot head with social cues vs. no embodiment (voice only via a domestic smart speaker). The purpose of our study was to investigate whether a robotic head mediator CA—with social cues—would benefit participants’ task performance relative to a disembodied smart speaker. Would there be a difference in participants’ trust perception? Would the embodiment increase engagement or be a distraction? Would there be an effect on the quality of the interaction and on the subjective impressions of the users? The aim of this experiment was to answer these questions.

The contributions are as follows.

1. Empirical evidence that the participants’ duration of gaze towards the embodied CA was significantly higher in the human-like robot condition than the smart speaker, thus indicating more engagement in the robot condition.
2. Indications that embodied CAs could potentially be trusted more than smart speakers in task-based interactions.
3. Qualitative insights into the perception of embodied CA co-workers. For the majority, these were positive, but a minority of participants found the humanness (e.g., the face) of the embodiment a little unsettling or distracting (Mori, 1970; Mori et al., 2012).

The rest of this paper describes relevant prior work, our study design, measures, experimental setup, and results. We discuss the significance of our findings in the context of previous work, the limitations of this study, and directions for future research. Finally, we conclude with a summary of our findings.

## 2 Background

As stated in Introduction, CAs, sometimes termed intelligent agents or virtual agents/assistants, have recently become popular in domestic settings as voice-only smart speakers. They have also been studied as visual virtual entities with avatar faces and full-body avatars or embodied as physical robots in other contexts (Cassell et al., 2000; Jokinen and Wilcock, 2014; Shamekhi et al., 2018). In this section, we give an overview of prior work relating to virtual or physical embodiment of CAs, the anthropomorphic design of features such as facial expressions, and the effects on the human–robot interaction, including general perception, user confidence, task performance, and trust. We show that these effects are often nuanced and context- or task-dependent. We discuss verbal and non-verbal behaviours, how these can have an impact on trust and task performance, and how individual differences in users can be relevant to how they perceive robot behaviours. Finally, we discuss how eye gaze and CL might be used to assess user engagement.

### 2.1 Robot embodiment and anthropomorphic design

Two decades ago, when investigating the effect of personification on the trustworthiness of an intelligent agent, Van Mulken et al. (1999) found that the degree of personification (text, audio, cartoon, or life-like video) did not significantly affect trustworthiness. More recently, embodiment was one of the five different design themes (social intelligence, voice or style of communication, embodiment, non-verbal communication, and performance quality) identified by Rheu et al. (2021) as affecting the user’s trust towards a CA. Seeger et al. (2017) identified two opposing theoretical viewpoints on the relationship between anthropomorphic design and user trust in CAs. One, the human–human trust perspective posits that increasing anthropomorphism in a CA will increase trust. The other is the human–machine trust perspective that humans trust computers more than they do humans, i.e., increased anthropomorphism will decrease user trust in a CA. Thus, the issue of whether CA embodiment will improve trust remains an open question.

Embodiment has also been found to be a factor affecting task performance and task load, also referred to as CL (Kim et al., 2020). With regards to assisting the user in their task, it has been shown that using a virtual assistant can improve task performance and that embodiment enhances this over voice alone. Kim et al. (2020) investigated task load and CA embodiment in a within-subject study. Participants performed a desert survival task in the following three conditions: 1) performing the task alone, 2) working with a disembodied voice assistant, and 3) working with an embodied assistant. The results showed that both assistant conditions led to improved performance over performing the task alone, but they reported task load with the embodied assistant to be significantly lower than with the disembodied voice assistant. The task was carried out with information cards and augmented reality (AR) visual and audio stimuli. The AR assistant had a body, facial expressions, and gestures. In an early study, Cassell et al. (2000) investigated the use of embodiment to improve interaction with CAs, such as turn-taking in a virtual embodied CA for an estate agent, and found that embodiment could lead to qualitative improvements in dialogue. These studies back-up the findings in our study, but where they use a virtual agent, we use a physical social robot.

In a study on the effects of embodiment and social behaviours in an intelligent assistant (including entering and exiting the conversation setting), Kim et al. (2018) found improvements in user confidence in the agent’s ability to influence the real world and to respect their privacy. This is an example of a study where the particular context and task are closely connected to the conclusions reached. Indeed, Shamekhi et al. (2018) investigated the effect on group social perception of a group facilitation agent in terms of rapport, trust, intelligence, and “power.” They found that the value of “having a face” depended on the types of assistance that the agent was providing. They also refer to studies that argue that embodying a CA is unnecessary (Van Mulken et al., 1999; Hasegawa et al., 2010; Häuslschmid et al., 2017) and other studies that show that embodiment can improve users’ subjective impressions of a CA (Takeuchi and Naito, 1995; Bickmore and Cassell, 2001; Gratch et al., 2007). Dehn and van Mulken (2000), in an early review study on the impact of animated interface agents, concluded that there was no evidence that they improved task performance. Yee et al. (2007) later had similar findings. On the other hand, Shamekhi et al. (2018) argued that for constant engagement tasks, such as tutoring, embodiment can help, but that these benefits may not extend to tasks requiring intermittent interaction.

As to what style of robot embodiment might be suitable for a workplace CA, the use of the Furhat^2^ robotic head robot with social cues for situated interactions in serious settings is a growing area of research, e.g., museums (Al Moubayed et al., 2012) and homes (Kontogiorgos et al., 2019). We decided not to select from popular humanoid robots such as NAO, as these might be considered less suitable for workplaces or with adults, due to being deemed cute and playful (Mubin et al., 2016).

### 2.2 Verbal and non-verbal behaviours

As dialogue and social cues are inextricably linked, verbal and non-verbal behaviours are an important aspect of any embodiment study. The verbal behaviour of adding small talk to a conversation was investigated by Bickmore and Cassell (2001) in relation to its effect on trust (Bickmore and Cassell, 2001). In a study with an embodied virtual agent with two conditions, small talk vs. purely task-oriented dialogue, they found that while there was no overall main effect of the condition on trust, there was an interaction effect between user personality and trust. Small talk had no effect on introverts’ trust but did have a significant effect on extroverts’ trust. For extroverts, trust was higher with small talk than with purely task-oriented dialogue. So, we can see that the trust of users to whom these behaviours appeal, at least, can be influenced by the social behaviour of an embodied CA. Although we keep the verbal behaviours the same in our study between conditions, this prior work illustrates that trust is highly subjective, and verbal behaviours can be perceived differently from person to person.

With regards to non-verbal behaviours, humans are highly sensitive to the gaze of others. In a study with a Furhat robot (as we use in our study), Zhang et al. (2017) showed that participants solved quiz puzzles much faster when interacting with a Furhat robot that used eye movements for mutual gaze than in a non-eye movement condition. Hemminahaus and Kopp (2017), in another work, developed an adaptive system also using a Furhat robot that adjusted its use of gaze, facial expression, head gesture, and speech to guide participants to solve a memory puzzle. They demonstrated that by adapting these behaviours, task performance could be improved.

### 2.3 User engagement, gaze, and cognitive load

With regards to user engagement, we examine in our study both gaze and CL to establish engagement. Prior work has observed that an increase in gaze towards the robot or an agent is associated with high levels of user engagement during an interaction (Nakano and Ishii, 2010; Admoni and Scassellati, 2017; Ahmad et al., 2019b). Gaze has also been used to express cognitive effort in human–agent (Andrist et al., 2013) and human–robot interactions (Andrist et al., 2014). CL has also been studied in the context of user engagement. It has been found that limited engagement with the task, causing boredom, results in the reduction of CL (Schweppe and Rummer, 2014; Sharek and Wiebe, 2014; Padgett et al., 2019). In the context of the relationship between trust and CL, prior work has mostly focused on high workload and trust, finding a negative correlation between these two variables (Biros et al., 2004; Oviatt et al., 2004; Samson and Kostyszyn, 2015; Ahmad et al., 2019a). Previously, CL has been measured using the subjective one-off measure of NASA-TLX following completion of a task (Hart, 2006). More recently, however, it has been shown that CL can be monitored online during a task through non-invasive physiological measurements including pupil diameter (Ahmad et al., 2020a; Ahmad et al., 2020b). It should be noted that workload or CL has not been widely studied in HRI studies (Hancock et al., 2011; Hancock et al., 2020). The topic is more often found in the human–automation interaction literature. Nonetheless, studies using self-report measures of CL or those causing variation in the amount of CL in different contexts have been attributed to affecting trust in HRI systems (Chen et al., 2016; Ahmad et al., 2017).

In this paper, we use both gaze and CL to monitor engagement. We use gaze to ascertain users’ focus of attention over time. We use CL as a continuous measure to understand when users vary in CL, thus indicating varying engagement.

## 3 Materials and methods

### 3.1 Study design

#### 3.1.1 Research questions

Trust is affected by the amount of risk induced by the task and how much the user feels vulnerable being in the situation (Hancock et al., 2020). The originality of the work lies in the interaction design. The study (which we describe in the following section) enables the participant to rely on a mediator robotic interface (Furhat v. s. speaker) to carry out operations by controlling robots in an emergency situation simulation. The underlying situation places participants at the risk of failing the mission, making them vulnerable and presents an opportunity to study the relationship of the embodiment of the mediator robot with participants’ trust, engagement, satisfaction, and task performance.

To the best of our knowledge, the effect of embodiment in a CA has not been investigated in this way before.

Considering the research gap and dynamics of the experimental task, we formulated the following research questions for our study.

• RQ 1: Is user trust in a mediator CA affected by its embodiment?
• RQ 2: Is user engagement during a task affected by agent embodiment?
• RQ 3: Is user task performance affected by agent embodiment?
• RQ 4: Is the quality of interaction in terms of user satisfaction and the subjective impressions of users, affected by embodiment?

#### 3.1.2 Conditions

We used a repeated measures design with two conditions.

**FIGURE 1:**
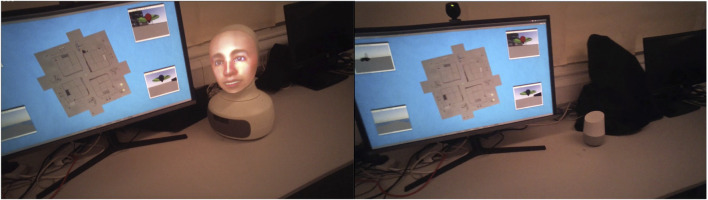
Two conditions captured from participants’ eye-tracking video feeds (uncropped). On the left is the robot located beside the display screen. On the right is the speaker similarly located.

Robot condition: The CA is embodied as a Furhat robot with social cues, including an expressive face with face, eye, and head movements (the face being a back-projection). The robot shifts gaze between the display and the participant. We used a male voice and face (this choice being described in the following section). To ensure consistent visual presentation, the light levels in the room were held constant and checked with a light meter at the start of each session (see Figure 1, left).

Speaker condition: The CA is embodied in a Google Home smart speaker, acting simply as a speaker for our dialogue system, providing voice-only interaction. Both conditions used the same text-to-speech synthesis and voice as described in the following section (see Figure 1, right).

As much as possible, we aimed to keep the interaction the same between conditions. The dialogue management for both conditions used the Wizard of Oz (WOz) paradigm, a method used in other CA studies, e.g., Bickmore and Cassell (2001), where an experimenter (the “Wizard”) simulates the behaviour of a theoretical intelligent computer application (by intercepting all communications between participant and system, often concealed or behind a blind). The participant is, typically, unaware of this until a post-experiment debrief (Kelley, 2018). We used a system similar to that used by Lopes et al. (2019) and Lopes et al. (2020). An experimenter (the Wizard) operated the dialogue manager through an interface, which facilitated the scenario conversation through an interaction flow based on a finite-state automaton, where each state represents a dialogue state. For each state, a number of hand-crafted prompts, some of which are populated by state-specific data such as robot names and timings, are automatically uploaded ready for selection by the Wizard, thus enabling a fluent dialogue closely tied to the simulation context. This method expedites the Wizard interaction and also constrains what dialogue actions the Wizard could perform in various contexts, thus keeping the interaction consistent across both conditions. As we were not investigating the effect of CA voice on how participants perceived their interactions, we kept this constant between conditions. Prior work has shown that the gender of a CA voice may affect user preference and whether or not users identify with the agent (Edwards et al., 2019). For example, Chang et al. (2018) concluded that users preferred a female-voiced CA as is often deployed in digital assistants or call centres. Other works have shown that users prefer interacting with a robot whose voice reflects their own gender (Eyssel et al., 2012). There has also been work showing that people tend to report more cognitive trust towards a male as opposed to a female human-like robot (Ghazali et al., 2018). However, we needed to rule out the assistant’s voice as a factor between our two conditions, and therefore, a male voice was used in both experimental conditions to avoid any confounding factors^3^. All of our participants would experience that same voice in both conditions.

#### 3.1.3 The task

Our aim was to challenge participants with managing a remote team of mobile robots in a simulated high-stakes emergency response scenario, set in an off-shore energy platform environment to help them become involved and engaged in their task (Ahmad et al., 2020b). To stimulate a sense of jeopardy and urgency, a time limit was set for managing the resolution of the emergency. The aim was that participants would feel a sense of urgency to make decisions quickly, thus increasing the opportunity for CL, and motivation to take the initiative in requesting information and, thus, stimulate the interaction. Due to the realistic simulation and task setup, the users were motivated to complete the task and were acutely aware of the time limit as it approached.

The task to be performed by the participants was to manage, in simulation, a team of mobile robots, including two drones, and two wheeled robots (Huskies), in a scenario simulating a fire breaking out on part of a fictional offshore platform consisting of four towers each designated by points of the compass (Figure 6). The simulation adapted the work by Pairet et al. (2019) and was carried out in Gazebo^4^ and controlled by the experiment system via ROS^5^. Each scenario was initiated by the mediator CA introducing itself and then announcing that there was an alarm in a particular place on the platform. The mediator CA informed the participant that there were a specific number of minutes within which they would need to resolve the emergency or an evacuation would be automatically commenced. After this, the dialogue continued with mixed initiative. The mediator CA would announce key facts, such as the results of surveillance or that the fire had been extinguished, and the participant could ask their own questions or make decisions when prompted by the mediator CA. The interaction ended when the fire was extinguished and a damage survey report had been obtained or when the time limit was reached without the fire being extinguished. This sequence of interaction periods is set out in Table 1.

**TABLE 1:** Interaction periods: Interactions consisted of up to three periods, where the emergency was successfully resolved (i.e., when a remote robot reached the fire location and activated its extinguisher), and the interaction involved all three periods. An interaction in which the emergency was not successfully resolved did not include the damage survey; instead, it ended after a time limit during the extinguish fire period.

	Interaction period	Description
1	Inspect	Inspect the emergency location
2	Extinguish	Attempt to extinguish the fire (time limited)
3	Survey	Survey the damage (only if fire resolved)

### 3.2 Measures

In the following, first we categorise our measures (objective, subjective, and qualitative) and then describe specifics about the measures and how they were gathered.

#### 3.2.1 Categories of measures

The data collected from participants fall into three categories: objective measures, subjective measures, and qualitative data.

The objective measures were gathered via eye-tracking video stream, the interaction manager, and interaction audio recordings. CL and gaze were gathered using eye-tracking glasses and together were used to monitor user engagement, as described at the end of Section 2. Last, interaction-related data were gathered through the dialogue/scenario manager (e.g., average turn duration) and also through transcription and analysis of audio recordings.

The subjective measures were trust, measured using the Schaefer 14-item trust scale (Schaefer, 2013), and user satisfaction, measured using a four-item scale adapted from the PARADISE evaluation framework (Walker et al., 1997).

The qualitative data were collected using a post-task questionnaire, which prompted participants to contrast the two conditions.

#### 3.2.2 Details of the measures

Here, we give specifics about our measures structured by research question.

##### 3.2.2.1 RQ1—trust

Participants completed a trust questionnaire after each condition. We used the 14-item trust scale (Schaefer, 2013), which yields a score free to vary from 0 to 100 and is continuous data representing user trust in the robot. This 14-item scale consists of a subset of items from, and positively correlating with, the 40-item scale by the same author. We opted for the 14-item scale so as to reduce participant fatigue.

##### 3.2.2.2 RQ2—user engagement (using CL and gaze)

To assess engagement, we examined both CL and gaze. We detail how these were gathered in the following.

CL was measured by processing the video stream from Tobii eye-tracking glasses (worn by participants throughout the experiment) (Figure 5), extracting pupil diameter change (this provides continuous physiological monitoring throughout the task as opposed to a post-task self-report such as NASA TLX (Cao et al., 2009)).

It has been shown that measurements of the change in a person’s eye pupil diameter can be used to model CL, and we used the method described by Ahmad et al. (2020b,a) and Hennings et al. (2021) to measure and compute the mean pupil diameter change (MPDC) for each participant, throughout the interactions. To account for different pupil sizes, we extracted the raw data for both eyes. We applied three steps to clean the data. First, we removed the instances in the raw data on pupil size for the left and right eyes, where the sizes contained negative values. Then, we applied three filtering methods, one after another, to filter the data. These were: 1) dilation speed outliers and edge artifacts, 2) trend-line deviation outliers, and 3) temporally isolated samples as described by Ahmad et al. (2020a) and Kret and Sjak-Shie (2019). Finally, we performed data sectioning and conducted our analysis.

We divided up each participant interaction into its component periods (see Table 1 for a description of this and how the three period labels were derived). To compute MPDC, we calculated the ratio between the overall mean of pupil diameter (PD) in millimetres over all the three periods together (i.e., *inspect*, *extinguish*, and *survey*) and the mean of PD in millimetres during each of the individual periods of the interaction for both the left and right eyes, as described by Ahmad et al. (2020a). As MPDC represents a ratio, normalised for each participant across one of their interactions, it has no units. Last, we averaged the MPDC measurements for the left and right eyes to produce a single measure representing normalised CL for each participant.

Gaze was manually annotated from the eye-tracking video by noting the timestamps when the participant’s gaze was on the agent (robot or speaker).

Together, CL and gaze were used to monitor user engagement as described at the end of Section 2.

##### 3.2.2.3 RQ3—task performance

Interaction-related data, as stated previously, were gathered through the dialogue/scenario manager and also through manual and automated transcription of the interactions from audio recordings: average turn duration, average number of words per turn, number of system turns, number of user turns, user and system time between turns, time on task, and whether or not the emergency was resolved.

This also included calculating the time taken for participants to respond to system requests for decisions, which we term planning time.

Gauging task performance: The level of reality in the scenarios within the simulations was a compromise among realism, providing variety, and limitations within our simulation. On the side of realism was the speed of movement of the remote robots (drones and Huskies). The drones were faster, and the Huskies were slower. On the side of providing variety, we allowed the possibility that any remote robot, be it a drone or a Husky, could be used for extinguishing fires or surveillance. A limitation of our simulation was that when two robots of the same type were tasked consecutively by the participant, the first had to return to its base before the other moved to avoid collisions. Along with the time limit used to inject urgency and a sense of jeopardy into the interaction, these aspects meant that whether or not the fire was extinguished before the time limit ran out was largely a factor of which robots a participant selected to be used. Thus, it was not a suitable measure of task performance. We looked, instead, at how quickly participants responded to the request by the mediator CA for a decision on which remote robot to use at different stages of the interaction, e.g., surveillance to inspect the emergency area, or for fire-fighting. We describe this in the following as planning time.

Planning time: The amount of time it took for participants to respond to the CA’s request for a decision on which robot to use for inspection or action was calculated and averaged for each participant across all such decisions in an interaction. The episodes of planning time were gathered by manual transcription of the audio of each interaction using the Praat^6^ audio wave-form segment labelling tool to produce accurate time-stamped transcripts, which were then analysed to extract the response times.

##### 3.2.2.4 RQ4–user satisfaction and subjective impressions

User satisfaction: After each condition, participants completed a user satisfaction scale, adapted from the PARADISE framework (Walker et al., 1997), for evaluating spoken dialogue systems, consisting of the following four items: “It was easy to find what to do at each stage of the interaction,” “The personal assistant behaved as I expected,” “The personal assistant was often quick to answer to my requests,” and “I would use a personal assistant such as this in the future” all answered on a 5-point Likert scale (1 = “strongly disagree” to 5 = “strongly agree”). Each participant’s responses were treated as a scale, i.e., averaged and then analysed as continuous data in the range 1.0–5.0.

Subjective impressions: We carried out quantitative analysis of closed question responses and qualitative analysis of the open question responses to our post-task questionnaire.

Quantitative analysis of the questionnaire: After completing the experiment, participants answered a set of questions probing their preference from the interaction A) “*Which interaction did you prefer?*“; B) “*Which personal assistant do you think was the better of the two for the task of helping manage the emergency scenarios?*“; C) “*Which personal assistant do you think you would trust more in an emergency scenario?*“ Single-choice answers were supplied from the categories “Robot,” “Speaker,” and “Neither.” (It should be noted that these were presented to participants as “First, Second, or Neither” with “First and Second” being decoded when the balanced presentation order was unwound in analysis). The counts of participant responses were tallied, and a chi-square analysis was used to compare them.

Qualitative analysis of the questionnaire: Participants were also asked to give open responses and the reasons for their answers given in each of the aforementioned closed questions. The questionnaire ended with “*If you have anything else to say about the two personal assistants please comment here.*” These four extended responses were thematically analysed, by a single coder, using open coding and an inductive approach (Strauss, 1987; Corbin and Strauss, 2008). Participants referred to the conditions by the labels, “First” and “Second,” which were decoded during analysis as “Robot” and “Speaker” for each participant.

### 3.3 Experimental setup

#### 3.3.1 The apparatus

**FIGURE 2:**
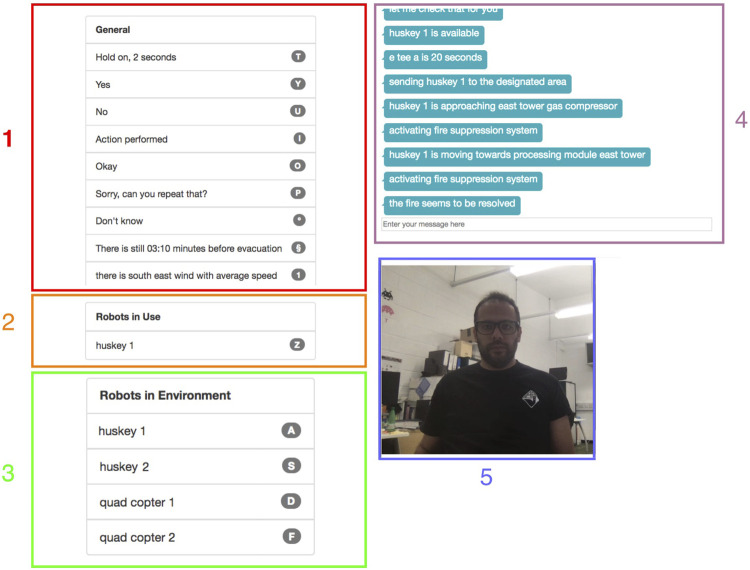
WOz interface. (1) Prompts available, (2) active robots, (3) available robots, (4) chat window, and (5) view of the participant.

The user performed a task based on a scenario simulated in an ROS Gazebo-based simulation, run on a graphics-capable laptop, and displayed on a 32-inch 4K display. The WOz interface and dialogue manager ran on a separate laptop. The WOz interface allowed the Wizard to visually monitor the participant through a webcam facing the participant on the simulation display (Figure 2). The Wizard could hear what the participant said and could quickly choose appropriately contextualised system utterances from a list in the WOz interface populated by the dialogue manager finite-state automaton. The available utterances included task- and state-based responses, such as how long it would take one of the mobile robot team to move to a particular location within the simulation.

The following devices were used for the two conditions: robot, a Furhat social robot from Furhat Robotics (Al Moubayed et al., 2012) (Figure 1, left); speaker, a Google Home (Figure 1, right) to the right of the display screen.

**FIGURE 3:**
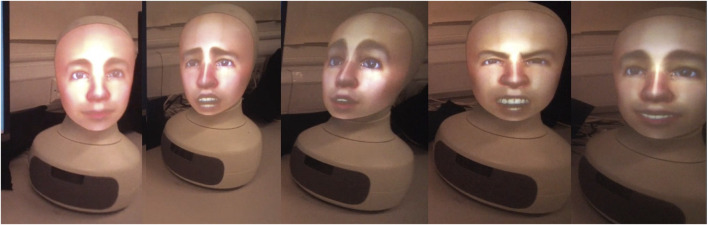
Facial expressions of the robot captured from participants’ eye-tracking glasses video feeds (cropped). From left to right: neutral, fear, sad, frustration, and happy. The neutral expression was the predominant expression displayed throughout the interaction. Momentarily though, very briefly, when specific events occurred, one of four other expressions was presented (see text).

**FIGURE 4:**
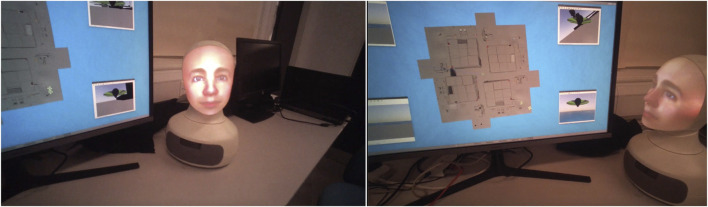
Robot head movement: Focussing on the participant to allow mutual gaze (left) and focussing on the display to cue shared attention when the robot was not talking or awaiting a response from the participant (right). These views are captured from participants’ eye-tracking video feeds (uncropped).

**FIGURE 5:**
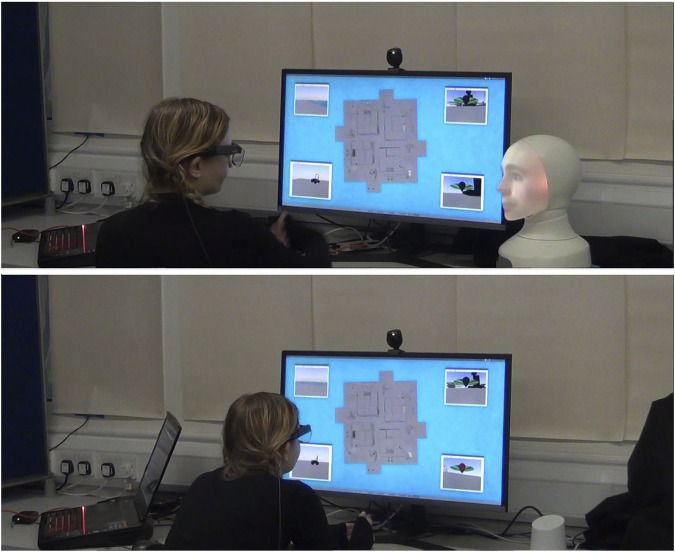
Participant (wearing eye-tracking glasses), display, and assistant. Condition 1- in this case, robot (top); condition 2- in this case, speaker (bottom).

The social cues deployed in the robot condition were as follows: we used the expressions fear (when an alarm went off), sad (when a participant request could not be fulfilled such as a particular robot not being equipped for fire-fighting), frustration (when the timeout was reached and the emergency was not resolved), and happy (when the emergency was resolved before time ran out) (Figure 3). Furhat would gaze at the participant when addressing them and would gaze at the screen when the remote robots were in action and there was currently no verbal interaction (Figure 4). The robot position was kept constant between participants, and the head movements (in degrees of turn and tilt in the horizontal and vertical planes) were also constant. Aside from embodiment and the social cues, there were no other differences in the two CAs. The same male voice was used for both conditions (as described earlier in Study Design). The participant seat position in relation to the display and assistant (robot or speaker) was kept constant although participants were free to lean forward or back as they wished (Figure 5).

#### 3.3.2 The three experiment phases

Each participant experienced the simulated task environment in three phases, which we describe as follows.

**FIGURE 6:**
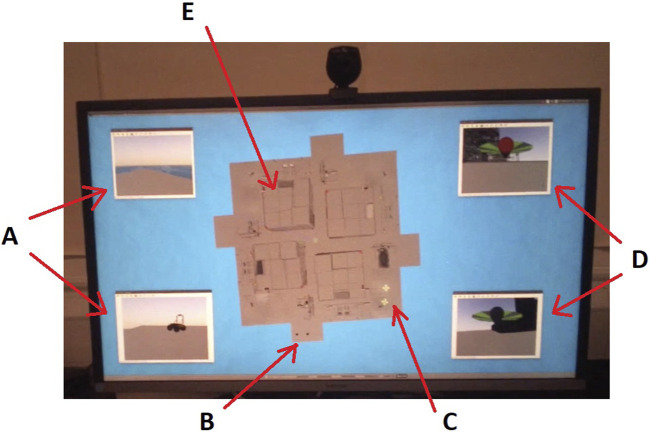
Simulation display as captured from a participant’s eye-tracking glasses video stream (cropped) showing the offshore installation top–down plan view in the centre, with the robot point-of-view (POV) cameras arranged in each corner. The labelled features are as follows: **(A)** POV camera views from the two Husky robots, one is looking out to sea, while the other is looking at its partner Husky, **(B)** two Husky robots on the plan view indicated by small rectangular models shown here in their starting locations, **(C)** two quadcoptors also in their starting locations, **(D)** POV camera views from the two quadcopters, both facing different parts of the installation superstructure, and **(E)** one of the four towers of the installation superstructure as viewed from above.

First phase—familiarisation: This was carried out to familiarise the participant with the task and the information flow during the emergency response scenario, which contained similar elements to the interaction scenarios to be undertaken in the two conditions, ensuring all participants started with the same base knowledge. The simulation display depicted a top–down plan view of the simulated off-shore installation, in which two drones and two wheeled robots moved in response to decisions made by the participant through the CA. In each corner of the display were arranged camera views of each remote robot’s point of view within the simulation (Figure 6). To avoid biasing either of the conditions, an experimenter took the role of the mediator CA during the familiarisation phase and followed a script, first to explain the visual elements in the simulation and then to talk the participant through the scenario, prompting them for decisions in a similar way to the prompting they would receive during the two conditions by the CA. This aspect of the protocol was modelled on the study by Kontogiorgos et al. (2019) involving a CA-guided food preparation task (Kontogiorgos et al., 2019). Participants were told that there was a fixed time to resolve the emergency and they needed to establish the capabilities of their robot team through dialogue. Participants were told that they would have a personal assistant during the interactions. During familiarisation, the assistants (robot and speaker) were kept hidden under black felt cloth covers.

Second phase: Condition 1—either robot or speaker: The order of the condition was balanced across participants. The participant managed an emergency response scenario by interacting with the mediator CA embodied as either the robot or smart speaker.

To avoid biasing their judgements, at the start of each condition, as the participant was being seated, they were simply told that the personal assistant located beside the display was their personal assistant for the upcoming interaction. While not in use, each personal assistant was kept hidden from view beneath a black felt cloth cover. Between phases, while participants were seated at a different station a few feet away and facing away from the interaction station to complete questionnaires, the appropriate assistant was uncovered and moved into position in the assistant location for interaction. (A marked spot beside the simulation display kept this position constant between conditions and between participants.)

Third phase: Condition 2—the other condition with a different scenario of similar format but with the emergency located in a different part of the off-shore platform: The average duration for each interaction in both conditions was 4 minutes.

#### 3.3.3 Participants and ethics

We used email publicity and convenience sampling to recruit participants from our institution. Participation took about 45 min, and participants were compensated with an $18 shopping voucher. Ethical approval for the study was obtained from our institution.

There were 31 participants (16 male, 14 female, and 1 preferred not to say), aged between 18 and 43 (M = 23.7, SD = 5.0). With the exception of one non-student (a graduate), all were graduate or postgraduate students.

To gauge what experience and exposure participants had with CAs, we asked participants to list personal assistants they had used. The following were listed: Siri (mentioned 14 times), Alexa (11), Google Assistant (11), Cortana (8), and Google Home (5). On average, participants listed 1.7 assistants (Min, 0; Max, 4). They categorised the frequency with which they used a personal assistant: daily (N = 8), weekly(6), monthly (2), rarely (10), and never (5). Thus, half the participants used personal assistants only infrequently or never, and only 8 out of 31 used them on a daily basis.

#### 3.3.4 Experimental procedure

1. The participant was fully briefed (omitting the fact that the CA was operated by a Wizard, as is the procedure for WOz studies) and was asked to sign a consent form. The explanation included that an experimenter (in fact, the Wizard) would sit out of sight behind a screen and manage the recording of the data.
2. The participant donned Tobii Pro 2 eye-tracking glasses and a lapel microphone and underwent calibration of the glasses for CL measurement, watching a blank screen vary in brightness, as described by Ahmad et al. (2020a).
3. Familiarisation phase: See “The three experiment phases.”
4. The participant completed a demographics questionnaire including previous experience with digital personal assistants.
5. Condition 1: The participant sat in front of the simulation display, with either robot or speaker as the mediator CA beside the display, and managed an emergency response scenario with a fixed time limit to extinguish a fire^7^. (The order of condition was balanced across participants).
6. The participant completed the trust and user satisfaction items for condition 1.
7. Condition 2: Like condition 1 but the other condition and managing a similar scenario with a different location for the emergency. (The presentation of the two similar scenarios with different starting locations was balanced between conditions).
8. The participant completed trust and user satisfaction items for condition 2 followed by a post-experiment questionnaire prompting them to qualitatively contrast the two conditions. They were prompted to express preferences through three questions (see Section 3.2.2 RQ4), with options, “First, Second, or Neither” (later translated to robot, speaker, or neither in analysis accordingly), and asked to give their detailed reasons for each answer^8^.
9. Participants were thanked for participation and debriefed about the WOz aspect of the experiment.

## 4 Results

We report our results in the following referring to our research questions. Later, in Section 5, we interpret the significance of these findings.

### 4.1 RQ1—trust

“Is User Trust in a Mediator CA affected by its embodiment?”

The means of the 31 participants’ trust scores from the two conditions were not statistically significantly different. A paired-sample *t*-test was conducted: robot M = 83.55, SD = 12.0, speaker M = 83.02, SD = 13.6, *t* (30) = 0.367, and *p* = .716 (two-tailed).

A further result on trust is reported as follows in RQ4 (user satisfaction and subjective impressions) with other results from the post-task questionnaire. One of the items in the questionnaire asked participants a more focused question than those in the broader Schaefer 14-item trust scale (Schaefer, 2013), “Which personal assistant do you think you would trust more in an emergency scenario?“ A chi-square analysis of the observed counts of responses (reported in detail in the RQ4 results) indicated that users trusted the robot more than the speaker when prompted to contrast the two conditions with respect to an emergency response context.

These two results are discussed further in Discussion.

### 4.2 RQ 2—user engagement (using CL and gaze)

“Is User Engagement during a task affected by agent embodiment?”

In this section, we report on engagement, which we monitor using CL and gaze. We discuss each of these in turn.

#### 4.2.1 CL

**FIGURE 7:**
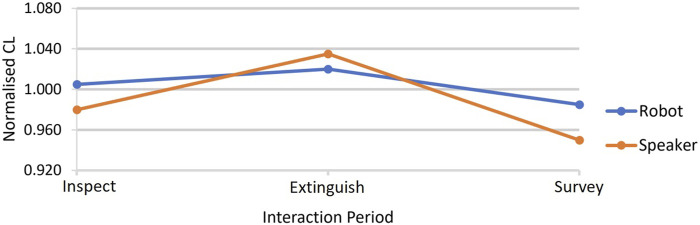
Normalised CL medians for the two conditions over the three periods of the interactions. Table 2 shows that when the distributions for each condition are compared by Wilcoxon tests, during the inspect period, CL was statistically significantly higher in the robot condition than for the speaker, but in the other two periods, the conditions were not statistically significantly different. Table 3 describes the statistical tests comparing CL between interaction periods within each condition. These tests show that CL did fall after the extinguish period during the survey period for both conditions (see text for interpretation of this).

We analysed the normalised CL measure for each interaction across all three periods (readers are reminded of the interaction periods described in Table 1). The results showed that during the *Inspect* period, there was a statistically significant effect of embodiment on CL. Interacting with the robot led to higher CL than with the speaker. When comparing between conditions, it was only in the *Inspect* period that a significantly higher CL for the robot than for the speaker was observed (see Table 2 and Figure 7). However, within each condition, statistical tests showed that, for both robot and speaker conditions, CL fell after the extinguish period when participants entered the survey period (see Table 3). We interpret this as being due to participants who were successful in extinguishing the fire against the clock in the extinguish period, for either robot or speaker condition, relaxing a little in the survey period because they no longer faced a time limit while managing the damage survey.

**TABLE 2:** Normalised CL descriptive statistics and test results for the two conditions over the three periods of the interactions.

Interaction period	Inspect		Extinguish		Survey	
Condition	Robot	Spker	Robot	Spker	Robot	Spker
**Median**	1.005*	**0.980**	1.020	1.035	0.985	0.950
Mean	1.028	0.988	1.045	1.031	0.980	0.964
SD	0.085	0.042	0.100	0.039	0.073	0.073
Max	1.305	1.080	1.390	1.110	1.085	1.070
Min	0.905	0.890	0.945	0.925	0.820	0.870
N	29	29	29	29	20	17
*p* (two-tailed)	**0.039**		0.808		0.545	
*Z*	**−2.056**		−0.252		−0.663	
*r*	**0.270**		0.033		0.161	

See Table 1 for a description of the interaction periods. The *Z* statistic shown is *Z* (CL speaker–CL robot). *r* is the effect size showing that during the inspection period the robot condition led to a small but significant increase in CL compared to the speaker condition (*significantly higher median). The data were not normally distributed, hence the use of the Wilcoxon signed rank test (Field, 2009). N indicates the number of interactions [not all interactions had a survey period (see Table 1)].

The emboldened values highlight the statistically significant pairwise comparison between Robot and Speaker conditions in the Inspect Period.

**TABLE 3:** CL for the different interaction periods was compared.

Condition	Interaction period pairing	*Z*-statistic	*p*-value 2-tailed (Bonferroni adjusted)
Robot			
	Inspect vs. Extinguish	−0.712	1.000
	**Survey vs. Extinguish**	**2.372**	**0.053**
	Survey vs. Inspect	1.660	0.291
Speaker			
	Inspect vs. Extinguish	−1.886	0.178
	**Survey vs. Extinguish**	**2.487**	**0.039**
	Survey vs. Inspect	0.600	1.000

Using the data described in Table 2, the three interaction periods’ normalised CL distributions were compared within each condition. For each of the two conditions, Friedman’s ANOVA (for non-parametric data) rejected the null hypothesis at the 95% confidence level, that all three periods were the same (robot: Friedman’s *Q*(2) = 6.000, *p* = 0.050; speaker: Friedman’s *Q*(2) = 6.939, *p* = 0.031). *Post hoc* pairwise comparisons by Dunn–Bonferroni tests were carried out, and these are shown in the table. Parings statistically significantly different (or near significant at the 95% confidence level) are shown in bold. See Figure 7.

The emboldened rows highlight the two statistically significant pairwise comparisons between Survey and Extinguish periods in both the Robot and Speaker conditions.

#### 4.2.2 Gaze focus

**FIGURE 8:**
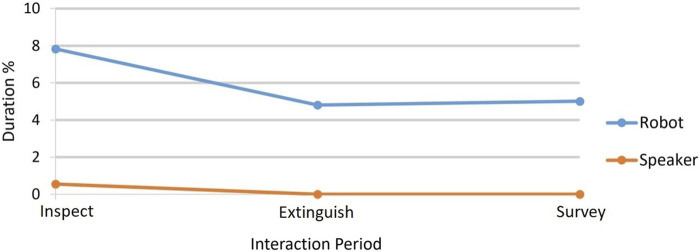
Gaze of participants towards robot vs. speaker represented by median percentage duration gaze directed at the agent during the three periods of an interaction. Table 4 shows that gaze towards the robot condition is statistically significantly higher than towards the speaker condition in all interaction periods.

Participants spent statistically significantly longer focussing on the robot and only rarely looked at the speaker. At the start of each interaction (during the inspect period), participants focused on the robot more than they did later, on average. This may have been due to a novelty effect but may also have been due to the inspect period involving more interaction with the CA introducing itself, giving information about an alarm being activated, asking if the participant wanted a PA announcement made and an early need for a decision on which remote robot to send to inspect the emergency location. Throughout the interaction, participants looked at the agent more in the robot condition than the speaker condition. As with the CL analysis, the interactions were divided by content into their sequence of periods (Table 1). The percentage of the time during an interaction period that each participant was focussing on the agent (robot or speaker) was calculated. These data are, therefore, normalised (0–100). Figure 8 shows the median percentage gaze duration for each interaction period for the two conditions. Table 4 shows the median, mean, SD, max, and min figures to accompany the chart. The results were not normally distributed, so Wilcoxon signed rank tests were carried out to compare the two conditions for the interaction periods. All were significant at the 95% confidence level, and there was a large effect of condition on the gaze percentage. See last three rows of Table 4. We also noted at a very low level, some gaze away from both the agent and the display, perhaps indicating inattention. These were noted as follows as percentages of each full interaction: robot, M = 0.373%, SD = 1.013%, Mdn = 0.280%, min = 0.000%, max = 4.900%; speaker, M = 0.774%, SD = 1.164%, Mdn = 0.280%, min = 0.000%, and max = 4.900%. A Wilcoxon test (Z = −2.483 *p* = .005, two-tailed) showed that this “inattention-gaze” was a significantly shorter duration for the robot than for the speaker.

**TABLE 4:** Percentage gaze duration result descriptive statistics and Wilcoxon signed rank tests comparing the two conditions over inspect, extinguish, and survey periods of the interactions.

	Inspect		Extinguish		Survey	
	Robot	Speaker	Robot	Speaker	Robot	Speaker
Mdn	7.834*	**0.555**	4.810*	**0.000**	5.009*	**0.000**
Mean	10.644	1.667	5.548	0.825	7.382	0.516
SD	10.973	2.377	4.709	1.834	8.167	0.853
Max	51.243	9.921	15.505	5.024	35.717	2.794
Min	0.339	0.000	0.00	0.000	0.000	0.000
N	29	29	29	29	20	17
*p*	< **0.001**		< **0.001**		**0.004**	
*Z*	**−4.465**		**−4.167**		**−2.521**	
*r*	**0.586**		**0.547**		**0.414**	

The *p*-values are two-tailed. *r* is the effect size [*r* = 0.5 represents a large effect (Field, 2009)]. *The significantly higher medians. See also Figure 8.

The emboldened values highlight the three statistically significant pairwise comparisons.

The gaze durations were annotated from the eye-tracking video data; however, the capture of this data failed for two participants; therefore, 29 were analysed.

Cognitive load and gaze focus: We note here that statistically significantly higher CL in the inspect period of the robot condition interaction (compared to the speaker) and the significantly higher gaze focus on the robot condition during the inspect period may be indicative of heightened task engagement with the robot condition.

### 4.3 RQ3—task performance

“Is User Task Performance affected by agent embodiment?”

There were no significant effects of the condition on any of the following tasks or dialogue-related measures: average turn duration, average number of words per turn, number of system turns, number of user turns, user and system time between turns, time on task, and whether or not the emergency was resolved. Therefore, for space reasons, we do not report the specifics of these.

Planning time: Participants took a statistically significantly shorter time to respond to the CA’s request for a decision on what robot to use in the robot condition compared to the speaker condition. See Table 5. This was a medium effect of the condition on the planning time.

**TABLE 5:** Planning time (in seconds) descriptive statistics and Wilcoxon signed rank test results.

Condition	Robot	Speaker
Mdn	4.375*	**4.992**
Mean	4.420	5.088
SD	0.620	1.750
Max	5.511	10.790
Min	3.358	1.513
N	29	27
*p* (two-tailed)	**0.008**	
*Z* (speaker–robot)	**−2.402**	
*r*	**0.321**	

Participants’ planning time was significantly shorter with the robot [*r* = 0.3 is a medium effect (Field, 2009)]. The speaker results were not normally distributed, so Wilcoxon’s test was used (*the significantly lower median). Due to technical reasons, one or both interactions for four participants (out of 31) failed to record (see row showing N).

The emboldened values highlight the statistically significant pairwise comparison.

### 4.4 RQ4—user satisfaction and subjective impressions

“Is the quality of interaction in terms of User Satisfaction and the subjective impressions of users affected by embodiment?”

In this section, we report the results from the user satisfaction scale items for each condition followed by the results from the post-experiment questionnaire. This questionnaire allowed a quantitative analysis of the closed response questions that we used to prompt the open responses, which were also subjected to qualitative analysis.

#### 4.4.1 User satisfaction

A paired-sample *t*-test exposed no significant difference in the scores for robot (M = 4.073, SD = 0.840) and speaker (M = 4.153, SD = 0.782) conditions; *t* (30) = −.648, *p* = .522, two-tailed.

Possible order effects (e.g., robot first vs. speaker first) on the user satisfaction rating were investigated. The Shapiro–Wilk tests of all ratings (*p* < 0.001), robot ratings (*p* < 0.01), and speaker ratings (*p* < 0.05) show that the ratings were non-normally distributed, so non-parametric statistical tests were computed. Wilcoxon signed rank square analysis showed that regardless of the embodiment being rated, participants rated the agent more favourably if they experienced the robot embodiment first than the Speaker embodiment (Mdn = 4.5 vs. Mdn = 4, *p* < 0.05). So, the robot had a positive influence on user satisfaction ratings when encountered first, and this positive effect was evident in the appraisals of both the robot’s user satisfaction and the speaker’s user satisfaction.

#### 4.4.2 Quantitative analysis of the questionnaire

**FIGURE 9:**
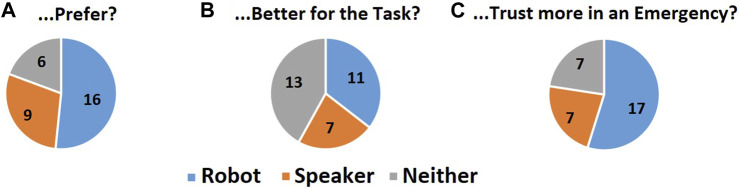
Pie charts of the counts of answers to the three preference prompting questions: **(A)** “Which interaction did you prefer?“; **(B)** “Which personal assistant do you think was the better of the two for the task of helping manage the emergency scenarios?“; **(C)** “Which personal assistant do you think you would trust more in an emergency scenario?” with three response options “Robot, Speaker, or Neither.” See the text for a statistical analysis of these counts and the detailed reasons participants gave for their opinions.

Chi-square analysis of the observed counts of responses to the closed questions revealed, for question A) “Which interaction did you prefer?,“ a near-significant preference for the robot (52% vs. speaker = 29% vs. neither = 19%), $\chi^{2}$ (2, N = 31) = 5.097, *p* = 0.078. There was, however, as mentioned previously in the RQ 1 results, a significant effect of embodiment on user trust as indicated by responses to question C) “Which personal assistant do you think you would trust more in an emergency scenario?,“ showing that, in an emergency scenario, users trusted the robot more than the speaker when prompted to contrast the two conditions (54% vs. speaker = 23% vs. neither = 23%), $\chi^{2}$ (2, N = 31) = 6.453, *p* < .05 (*p* = 0.040). No other trends were significant. The raw counts of these responses are shown in Figure 9.

#### 4.4.3 Qualitative analysis of the questionnaire

The qualitative themes from the responses of the 31 participants are set out are follows.

Theme 1—either comfort or uneasiness with the robot: Some participants expressed being comfortable communicating with the robot (N = 7), while others expressed unease (N = 4). Comfort: “*The human face/head makes it feel like you are speaking to more of a real person, there is something more reassuring about it*” [P30]. Unease: “*The [robot] was eerie due to human-like head …* ” [P26]. The speaker was only mentioned as giving comfort once and then in comparison with the robot. Specifically: “*The robot was more personal because it was a face but was a bit distracting and I’m more comfortable with the speaker as I use my Alexa on a daily basis*” [P20]. No participant expressed uneasiness about the speaker.

Theme 2—the robot face: Participants frequently commented about the robot face, human likeness, and how this affected their interaction. The following sub-themes are associated with this theme.

Sub-theme distraction/attention: A few (N = 4) participants described being distracted by the robot face. For example, “*I found the robot’s humanoid appearance distracting and unnecessary with respect to the needs of the mission*” [P14]. On the other hand, two indicated that it had enhanced their attention to the task, e.g., “*Because the face and eye movements can help 1*) *direct attention to the appropriate context and 2*) *enhance the sense that I am working with an assistant*” [P31]. These comments are in line with the quantitative results for user engagement, showing significantly more gaze directed towards the robot and significantly higher CL with the robot during the inspect period of the interactions.

Sub-theme trust: A few (N = 7) expressed that they felt their trust in the system was affected by the robot face. Some (N = 5) were reassured by the robot face and trusted it, P30 (quoted in Theme 1) being one example and P13, who commented, “*The interactive face makes it more sociable so, in a primitive human point of view, reliable,*” being another. Two participants distrusted the system because of the style of the face, e.g., “*If the face was more lifelike or animated better, [I] might trust it more as an AI*” [P3].

Sub-theme lifelikeness: A few (N = 7) commented on the degree to which the robot face was lifelike. Some were negative (N = 4) such as P3, quoted previously in the trust sub-theme.

On the other hand, some (N = 3) were positive, e.g., “*The robot personal assistant had a face, which makes it nicer to interact with and resemble more to a human-to-human interaction. [Then:] I think I can communicate better with “human-like” robot*” [P8].

Sub-theme positive and negative views: Many (N = 13) expressed positive reactions to the robot face, e.g., “*The robot one has facial interaction. It was easier to tell whether the personal assistant was processing or listening*” [P10], and “*…interaction was more pleasant with the simulated face movement*” [P9]. A few (N = 5) commented negatively about the robot face, e.g., “*…as it was looking more like a real person I felt a bit awkward talking to it*” [P26].

Theme 3 CA performance: Many (N = 10) expressed that they were swayed by their subjective perception of the speed and performance of one or other or both of the conditions, e.g., “*They both responded in similar timings and understood me [well]*” [P25]; “*It [the robot] seemed more responsive and gave more relevant information each time*” [P17]; and conversely, “*The speaker was faster and understood my instructions better*” [P6]. It should be noted that these were subjective impressions of individual participants. In fact, the only aspect about the CAs that differed between conditions across the experiment was the embodiment with social cues vs. voice only.

## 5 Discussion

In this section, we discuss our findings in relation to our research questions and prior work.

### 5.1 RQ1—trust

“Is User Trust in a Mediator CA affected by its embodiment?”

In this section, we discuss the two trust results, from the rating scale administered following each condition and from the post-experiment questionnaire item prompting participants to contrast the two conditions. We discuss possible reasons for the differing results.

We found no evidence of an effect of CA embodiment on user trust, as measured by the 14-item Schaefer rating scale in this context of managing a remote team of mobile robots through a mediator CA. This would agree with Van Mulken et al.’s (1999) finding in their study of the effect on perceived trustworthiness of different levels of personification of a virtual interface presentation agent which offered advice on navigating a web interface. That study framed the trustworthiness of an agent as depending on its competence and perceived predictability in terms of the advice it provides. They found that the degree of personification of the agent did not significantly affect participant ratings of agent trustworthiness. However, when we asked participants to contrast the robot and speaker conditions, we found a statistically significant result in the answers to question C) “Which personal assistant do you think you would trust more in an emergency scenario?” favouring the robot (Figure 9). We see three possible sources for these two differing results. They may be due to the difference between the processes of rating and ranking. We asked participants to rate with a trust scale, and at the point of rating, participants were anchoring their opinions in the interaction they had just experienced and the semantic stability of the rating scale, whereas with the post-experiment questionnaire, although its aim was to prompt qualitative comparisons, we were in effect asking participants to rank the conditions. It was not a forced choice (to avoid biasing the extended, qualitative, responses), and they were free to deem the conditions equal (by opting for *neither*), but most did still feel they wished to rank. In doing so, they were directly comparing the two conditions in respect to question *C*. Alternatively, the two results may reflect the multifaceted nature of trust, with the trust scale capturing broad trust and our question *C* being focused on, the perhaps narrower, trust in an emergency scenario. Moreover, to prevent participant fatigue, we used the short-form 14-item Schaefer scale as opposed to the 40-item full version (Schaefer, 2013), which may, possibly, have given different results where we have to use that full version. The 14-item scale was developed in the Schaefer thesis (Schaefer, 2013) and recommended by a panel of subject matter experts (experts in trust and robotics) as part of the development of the 40-item and 14-item scales. Comparing the performance of the two scales that work showed that the 40-item scale provided “a finer level of granularity and, thus, a more accurate trust rating.” In using the 14-item version of the scale, we were choosing to accept slightly less accuracy to reduce the likelihood of participant fatigue which might have been an issue where we have to ask participants to complete the 40-item scale for each condition. Given the various activities involved in each participant session, we still believe that a 40-item scale administered twice for each participant would have been too much, and the 14-item scale was a valid instrument to use in this repeated-measures experiment.

Qualitatively, the participant views expressed in our qualitative data fit with the thesis of Seeger et al. (2017) that there are two opposing viewpoints. For example, one participant (P30, quoted in Theme 1) expresses the “*human–human trust perspective*” (the more anthropomorphic the agent, the more trusted it is), stating that the human face and head made it more like a person and was reassuring. Another participant expressed the “*human–machine trust perspective,*” “*It [the speaker] looks like it’s part of a computer, and everyone trusts computers*” [P6]. Equally, these two viewpoints may reflect expression of the affective and cognitive dimensions of trust (Johnson and Grayson, 2005). Thus, in terms of user trust, our results serve to confirm prior work in some respects.

### 5.2 RQ 2—user engagement (using CL and gaze)

“Is User Engagement during a task affected by agent embodiment?”

Embodiment of the CA in a humanoid Furhat robot head with social cues led to significantly higher CL in the inspect period of the interaction scenario. The CL experienced by our participants, on the whole, during the interactions indicates medium levels of CL when compared to prior work using a similar method of in-task CL measurement (comparing the medians for MPDC in Table 2 with MPDC measurements in Ahmad et al. (2020a)). As we observe that they were not cognitively overloaded, we can postulate that the difference in CL is due to inactivity vs. engagement. There was also significantly greater participant eye gaze directed at the CA in the robot condition in all periods of the interactions. One way to interpret this, along with the medium levels of CL, is that the robot condition results in greater engagement in the simulated work environment as a whole, including both the task display and the robot coworker (Ikehara and Crosby, 2005). Indeed, during the task, the robot exhibits occasional joint gaze by looking at the screen with the participant and mutual gaze—behaviours similar to how co-workers might interact when working in front of a shared screen as indicators of social attention (Argyle and Dean, 1965; Breazeal and Fitzpatrick, 2000; Zhang et al., 2017). The observed engagement, both CL and eye gaze, was statistically significantly higher for the robot condition during the inspect period (Table 1 and Table 4). In this period of the task, participants were developing an appreciation of the situation and being asked for an early decision on which of the remote team to send to investigate the emergency. However, the CL results show that in the extinguish period, as the expiry of the time limit for extinguishing the fire approached, participant engagement in terms of CL was elevated for both robot and speaker when compared to the survey period, which followed it during which participants may have relaxed and become less engaged (see Table 3 and Figure 7). At the same time, eye gaze in the extinguish period, although still directed statistically more to the robot than the speaker, appears to have been less towards the CA than during the inspect period (Figure 8). This would make sense as during the extinguish period, the participants would be particularly interested in the progress of the remote robot moving to extinguish the fire on the display as the time limit approached. It is noteworthy that in qualitative *Theme 2*, sub-theme *Distraction/Attention*, two participants commented on the engagement encouraged by the robot, P1 stating, *“[With the Robot I] Felt it was easier to keep attention at the task”* along with P31 (already quoted in Section 5) stating that the robot face and eye movements helped direct attention. The significantly more inattention for the speaker condition noted in the small amounts of gaze away from both agent and display serves as further evidence that the robot increased engagement in the task and with the agent.

### 5.3 RQ3—task performance

“Is User Task Performance affected by agent embodiment?”

Embodiment of the mediator CA led to significantly faster responses by participants when asked for decisions. As far as the pace and flow of dialogue in the interaction was concerned, differences in speed of decision making were an interesting effect. This statistically significant medium effect leading to faster responses from participants when asked by the robot to decide which of the remote robots to use either to inspect the fire, extinguish the fire, or survey the damage (Table 5) would seem to be a beneficial effect in our context of a robot for managing emergency response. Clearly, more timely action is desirable when dealing with such situations in reality. Our robot condition used gaze behaviour, shifting its gaze between display and participant, and this may possibly parallel the performance improvements engendered by gaze cues as observed by Hemminahaus and Kopp (2017).

### 5.4 RQ4—user satisfaction and subjective impressions

*“Is the quality of interaction in terms of user satisfaction and the subjective impressions of users affected by embodiment?”* Initially, the lack of a significant difference in user satisfaction ratings between the two conditions would indicate no effect of condition on that measure. However, the order effect showing that the robot embodiment had a positive influence on user satisfaction ratings when encountered first is an interesting one. This positive effect of experiencing the robot embodiment first was evident in the appraisals of both the robot’s and the subsequent speaker’s user satisfaction ratings. It is interesting that despite designing the experiment as repeated measures and balancing the presentation order, the robot embodiment still manifested a positive effect of the robot on the user satisfaction ratings for the CA which carried over onto the speaker embodiment if that was experienced second.

The qualitative comments in, Theme 3, did reveal disagreement between some participants about whether one or the other performed better in terms of timeliness or in understanding their commands, despite there being no difference in the CAs other than embodiment. We do not have an answer to these perceived differences, some in favour of the robot and some in favour of the speaker. We were careful in the wording of our questions so as not to force participants into taking one position or the other. The questions clearly allowed participants to positively choose to state that they preferred, or trusted more, neither of the conditions. This was done deliberately so as to avoid confirmation bias affecting the qualitative reasons that participants might have expressed if the option had been forced choice (in favour of one or the other condition). That being said, there does remain the possibility that in instances where participants were finely balanced in their opinions between the two conditions they may have chosen to express a preference and gone on to be swayed by their own confirmation bias to express, as a reason, a subjective opinion that one condition’s responsiveness was better than the other condition.

Qualitative responses describing subjective impressions show that, while the robot condition was preferred by more participants (52%) (while not quite statistically significant, it was a trend), some viewed the two conditions even-handedly (19%) and some preferred voice-only interaction (29%) (see Figure 9, question A, and the themes in “Qualitative Results”). That some appreciated interacting with the robot head does fit with previous work presenting evidence that subjective impressions of a CA are improved by embodiment (Takeuchi and Naito, 1995; Bickmore and Cassell, 2001; Gratch et al., 2007). The minority of participants (4 out of 31) who expressed unease with the robot may perhaps have been experiencing the unease with human-like robots described by Mori (1970) and Mori et al. (2012). It has been shown that this does vary from person to person and is multidimensional (Paetzel et al., 2016a; Paetzel et al., 2016b; Paetzel, 2017). Indeed, there might be scope for implementing user customisation of an embodied CA robot face as suggested by Heuer (2019).

### 5.5 Limitations and future work

The use of a particular context and the chosen offshore energy platform simulation may be considered a limitation to the wider applicability of our results. However, given our wish to explore a serious work-based interaction, a realistic context was needed. Indeed, while our findings show evidence of these phenomena we describe in our results in this particular context, part of our contribution is in adding our context to the other studies with similar findings in a variety of contexts (Wang et al., 2018).

Given the subjectivity of the perception of embodiment, as discussed previously, and the fact that trust is also highly subjective and multi-dimensional, it is perhaps not surprising that not all of our outcomes in this experiment were statistically significant and, thus, do require further investigation. Furthermore, there is a possibility that there could be a novelty effect of the social robot affecting the interaction for our participant group who were, even allowing for the familiarisation phase in the experiment, non-experts. A study over time with users habituated to the conditions could be one way to investigate CAs in isolation from novelty. However, this aspect affects many HRI studies, and we believe it does not negate the contribution of our study in advancing the knowledge of how embodiment may affect perception and use of this genre of CA. Similar to many HRI studies, our participants experienced both conditions, robot and speaker, for the duration of the tasks (in this case, 4 min each on average across all phases of the interactions)^9^. This is not a longitudinal study where users have extended exposure to the conditions being studied. This is one factor why we only suggest that our results may indicate possible benefits of embodiment.

The Furhat embodiment while including head and eye movements, and facial expressions, does not include other body language such as hand gestures and body pose. Thus, the embodiment in this study carries that limitation.

The participants for the study were recruited from within our university rather than from within an industrial organisation that might deploy a remote robot team to manage emergency response on an energy installation. While they, perhaps, do not represent such an industrial employee pool, we believe they are approximately representative of the pool from which such an organisation might reasonably be expected to recruit job applicants.

Finally, we were careful in framing of the experiment to our participants and in the wordings of our questionnaire items to make sure that participants knew we were investigating the mediator CA and not the remote robots. However, there could be some fluidity between trust in the remote team and trust in the local mediator CA, in the minds of users. These remote robots’ behaviours were highly restricted, though, so across participants, there was little variation.

## 6 Conclusion

In a mixed-method study of the effect of embodiment on the use of a CA helping with a simulated but intense task, we measured participants’ trust, user satisfaction, and engagement; analysed interaction dialogue logs, audio, and transcripts; and gathered their qualitative impressions. While we found no significant effect of embodiment on our trust measure [the 14-item Schaefer scale, Schaefer (2013)], we did find statistically significantly more trust expressed towards the robot by participants when they contrasted the conditions in the post-experiment questionnaire. We also found that when participants experienced the robot condition first, this resulted in higher user satisfaction ratings for both the robot and the speaker conditions, thus showing a positive effect on this subjective impression of the CA when embodied as the robot.

Furthermore, we found that embodying the CA as a social robot head led to increased participant engagement, as demonstrated through cognitive load and gaze, particularly in the early stages of their interaction, compared to embodiment as a voice-only smart speaker device. In addition, participants’ decisions were faster when interacting with the robot condition, leading us to conclude that an embodied CA may be beneficial in high-stakes, time-critical applications.

This work has implications for voice assistants and robots in the workplace.

## Data Availability

The raw data supporting the conclusion of this article will be made available by the authors, without undue reservation.

## Footnotes

1 In a 2018 poll, 63% of Americans said they use voice assistants such as Apple Siri, Google Assistant, or Amazon Alexa, Kocielnik et al. (2018).

2
https://furhatrobotics.com/

3 The voice used was the only Amazon Polly text-to-speech British English male voice, and the robot face was the default male face available in the Furhat robot tools.

4
http://gazebosim.org/

5 The open source, Robot Operating System: http://ros.org/

6
https://www.fon.hum.uva.nl/praat/

7 Piloting the scenarios showed that allowing 4 min would be enough time to resolve the emergency and that participants who did not act fast enough or made sub-optimal choices would not resolve the emergency.

8 The “Neither” option was deliberately used to avoid forcing a choice as it was their reasons that we sought, and to force the choice might have caused them to introduce bias in their reasons towards one or other condition when no bias truly existed in their mind.

9 E.g., Kontogiorgos et al. (2019) used a task of 3–4 min duration for the conditions.
